# How is Artificial Intelligence Transforming the Skin Cancer Screening Pathway? An Umbrella Review

**DOI:** 10.21203/rs.3.rs-9069373/v1

**Published:** 2026-03-20

**Authors:** Lydia J. Sollis, Arianna Bunnell, Eujin Cho, Mark L. Willingham, Gabriela Cruz-Mattos, Anson Arii, Christopher Lum, Kevin Casse, John Shepherd

**Affiliations:** 1 Department of Epidemiology, University of Hawai’i Cancer Center, University of Hawai’i at Mānoa, Honolulu, HI, USA; 2 Research Corporation of the University of Hawai’i, Honolulu, HI, USA; 3 Complete Dermatology, Honolulu, HI, USA

## Abstract

**Background:**

AI algorithms for skin cancer detection have shown performance comparable to clinicians in controlled settings, yet their real-world reliability, performance across diverse populations, and readiness for clinical deployment remain uncertain. This umbrella review synthesizes evidence across the screening pathway to characterize AI performance, identify equity gaps, and assess implementation readiness.

**Methods:**

We searched PubMed, Web of Science, and CINAHL (November 6, 2024) for systematic reviews and meta-analyses evaluating AI for skin cancer detection, excluding narrative reviews, scoping reviews, and reviews not reporting diagnostic accuracy. Two investigators (LS, AB) independently screened studies and assessed quality using ROBIS; one (LS) extracted data with verification by a second (AB). Findings were synthesized narratively by screening phase. This study is registered with PROSPERO (CRD42024605934).

**Results:**

Of 411 records identified, 37 (2008–2024) met inclusion criteria; 10 (27.0%) were judged low risk of bias, 22 (59.5%) high, and five (13.5%) unclear. Self-screening applications demonstrated marked performance variability (sensitivity 0–98%), with reduced sensitivity for melanoma detection reported across reviews. Primary care AI achieved moderate accuracy (sensitivity 60–84%, specificity 88–93%). Specialist dermoscopy-based AI achieved sensitivities comparable to dermatologists (82–91%), and histopathology AI achieved 90% sensitivity. AI augmentation increased clinician sensitivity by 6–8 percentage points, with greater benefit for generalists (+28%) than specialists (+2%). Engagement with skin tone and ethnicity increased but remained largely superficial, and >70% of datasets were from light-skinned populations. Evidence disproportionately targeted melanoma (>40% of reviews) despite it comprising <2% of skin cancers; no reviews employed implementation science frameworks.

**Conclusions:**

Current evidence does not support unsupervised clinical deployment of AI-based skin cancer detection. Self-screening tools demonstrate inconsistent performance, equity gaps persist, and common non-melanoma skin cancers remain understudied. These findings support the need for stage-specific validation standards and performance reporting.

## Introduction

Skin cancer is the most common malignancy globally^1,2^, with squamous cell carcinoma (SCC) cases rising 310% between 1990 and 2017^3^ and annual U.S. treatment costs reaching approximately $8.9 billion as of 2018^4^. Early detection critically influences outcomes: localized melanoma has a 5-year relative survival exceeding 99%, falling to 35% post-metastasis^5^. These trends have driven efforts to advance screening and early detection methods^6,7^.

Traditional detection relies on clinical and self-examination guided by the ABCDE criteria^6,8^, supplemented by dermoscopy^6,9^, reflectance confocal microscopy (RCM)^10^, and total body photography (TBP)^9^, among other modalities. However, shortages of dermatologists, a lack of systematic screening^11^, and varying accuracy of lesion detection among generalist clinicians^12^ can limit access to care, particularly in rural and underserved areas^13^. Consequently, artificial intelligence (AI) algorithms have emerged as potential tools to augment diagnostic decision-making, as they have demonstrated diagnostic performance comparable to dermatologists in controlled settings^14,15^. Despite this potential, evidence regarding real-world outcomes such as biopsy reduction is mixed^16^. Successful integration of AI tools into clinical workflows also requires attention to implementation factors that are addressed through frameworks such as RE-AIM (Reach, Effectiveness, Adoption, Implementation, Maintenance)^17^ and CFIR (Consolidated Framework for Implementation Research)^18^.

Numerous systematic reviews have evaluated AI’s diagnostic utility for skin cancer, with the earliest inclusion-eligible publications dating back to 2008 and a substantial acceleration over the past decade. However, high heterogeneity in study designs, AI models, and validation methods, incomplete reporting on dataset diversity^16^, and an underrepresentation of diverse populations^19^ raise concerns about the generalizability of findings. This gap carries clinical consequences; melanoma survival is substantially worse among Black patients despite lower incidence^20^, skin cancers present differently across skin tones^21^, and AI algorithms have demonstrated reduced accuracy on darker skin^22^. Without representative training data and stratified performance reporting, AI tools risk perpetuating diagnostic disparities. Additionally, many reviews focus on specific phases of skin cancer screening, ranging from preclinical analysis to specialized imaging and biopsy-based diagnosis. A recent umbrella review by Karimzadhagh et al. (2025) synthesized 11 meta-analyses and confirmed high AI diagnostic accuracy^23^, but did not assess equity in training data, implementation readiness, or performance variation across clinical screening stages, and excluded systematic reviews providing qualitative evidence on deployment barriers.

**Figure 1:**
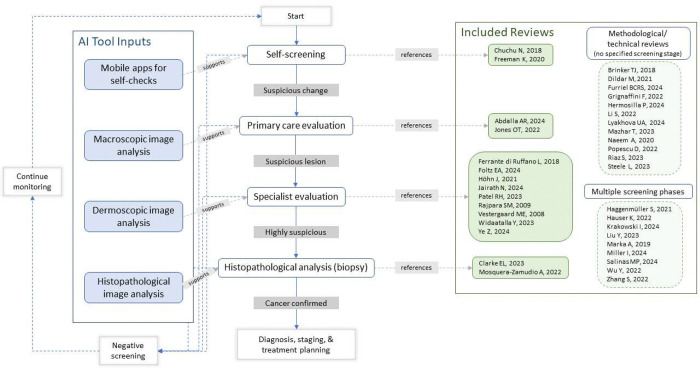
Screening pathway phases and corresponding AI inputs, with the number of included reviews mapped to each phase.

This umbrella review addresses these gaps by synthesizing evidence across four distinct care settings in the skin cancer screening pathway (Figure 1): (1) self-screening with mobile applications; (2) primary care evaluation with macroscopic clinical images; (3) specialist evaluation with dermoscopy or other non-invasive techniques; and (4) histopathological analysis of biopsy specimens. This umbrella review evaluates the diagnostic performance, population representativeness, and clinical implementation readiness of AI algorithms for image-based skin cancer detection across the patient care continuum. By synthesizing systematic reviews and meta-analyses spanning self-assessment through histopathological diagnosis, we aim to identify evidence gaps, assess equity in validation populations, and inform appropriate clinical deployment of AI-based screening tools.

### This umbrella review addressed three primary questions:

*Question 1: How does AI diagnostic accuracy vary across clinical care settings, and what is the effect of AI augmentation on clinician performance?* We assessed: (a) diagnostic accuracy of AI tools in self-assessment, primary care, specialist dermatology, and histopathology settings; (b) the impact of AI augmentation on clinician diagnostic performance; and (c) cancer types and imaging modalities with the strongest evidence.

*Question 2: How adequately do AI validation studies represent diverse populations, cancer types, and clinical contexts?* We examined: (a) the extent to which reviews addressed skin tone and ethnicity representation in training and validation datasets; (b) the characteristics, geographic distribution, and reuse patterns of publicly available datasets; and (c) alignment between evidence distribution across cancer types and clinical disease burden, including any reported performance disparities.

*Question 3: What barriers limit the translation of AI research into clinical practice?* We evaluated: (a) research question evolution and focus (algorithmic benchmarking vs. clinical implementation); (b) methodological characteristics including evaluation metrics, synthesis approaches, and quality of evidence; and (c) engagement with implementation requirements, including regulatory approval, prospective validation, real-world deployment, and use of implementation science frameworks (e.g., RE-AIM, CFIR) to assess factors influencing adoption and sustainability.

## Results

**Figure 2:**
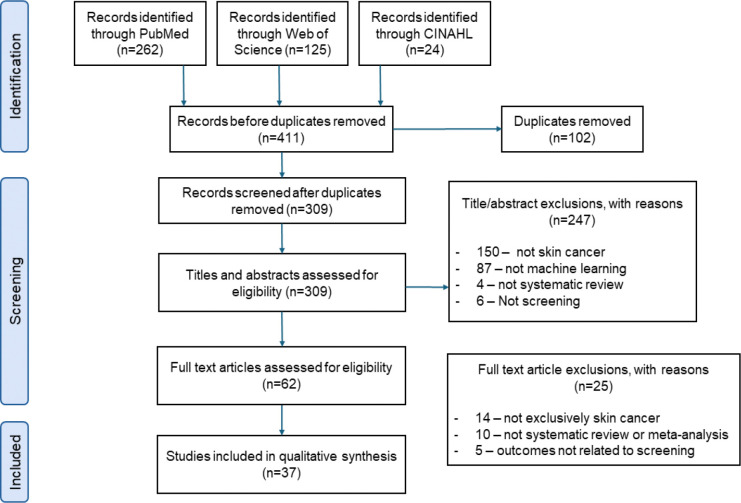
PRISMA 2020 flow diagram of study selection.

Our search yielded 411 records. After removing 102 duplicates and screening 309 titles and abstracts, we included 37 systematic reviews published between 2008 and 2024 (Figure 2). Publication activity accelerated substantially after 2019, with 31 of 37 reviews (83.8%) published from 2020 onward (Supplementary Figure S1).

Reviews addressed methodological or technical analysis (n=13, 35.1%), specialist evaluation (n=9, 24.3%), multiple screening stages (n=9, 24.3%), self-screening (n=2, 5.4%), primary-care (n=2, 5.4%), and histopathology (n=2, 5.4%) (Supplementary Figure S2). Overall, 10 reviews (27.0%) were judged low risk of bias, 22 (59.5%) high risk, and five (13.5%) unclear, with quality varying substantially by review type (Supplementary Table S1).

### Diagnostic performance across the screening pathway (Q1)

**Figure 3:**
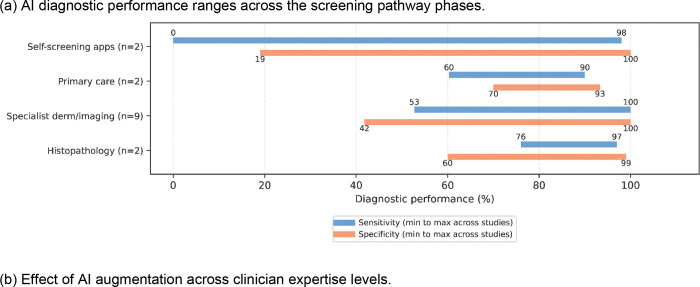

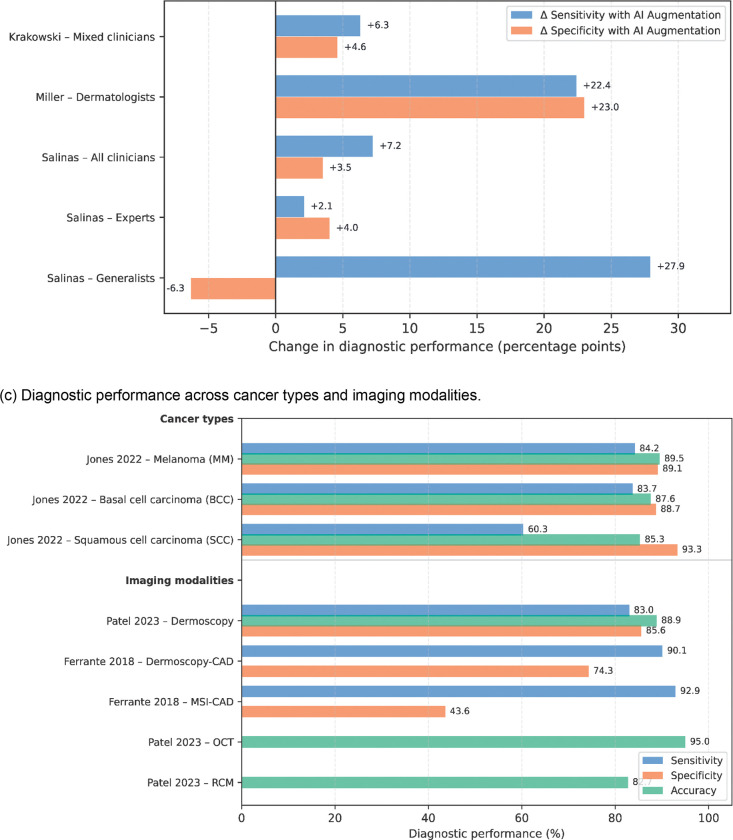
AI diagnostic performance across (a) the screening pathway phases, (b) clinician expertise levels, and (c) cancer types.

AI diagnostic performance varied substantially across screening pathway phases (Figure 3a).

#### Diagnostic accuracy of AI tools (Q1.a.)

Two reviews evaluated self-screening applications^24,25^, synthesizing evidence from 11 primary studies of commercially available smartphone applications using consumer-captured images. Both documented substantial performance variability (sensitivity 0–98%, specificity 19–100%), with frequent ratings of melanomas as unevaluable and concerns about applicability to real-world use. Both concluded that Al-based smartphone applications cannot currently be relied on to detect skin cancers.

Two reviews assessed AI for primary care^16,26^. Jones et al.^16^ synthesized evidence from 272 primary studies, finding only two synthesized evidence from low-prevalence settings reflective of primary care, with prospective validation conducted in approximately 2% of studies. Abdalla et al.^26^ identified only three studies meeting basic primary care criteria despite screening 473 records. Diagnostic accuracy was moderate to high: Jones found sensitivity ranges of 60.3% (SCC) to 84.2% (melanoma) and specificity ranging from 88.7% for basal cell carcinoma (BCC) to 93.3% (SCC), while Abdalla et al. reported 90% sensitivity and 85% specificity for suspicious pigmented lesions (SPLs). Neither review recommended widespread adoption without efficacy demonstration in low-prevalence populations.

Nine reviews evaluated AI in specialist dermatology contexts^27–35^. The evidence base was strongest for melanoma detection using dermoscopy, with meta-analytic sensitivity estimates of 82.0–91.0% and specificity of 74.3–91.0%. Ferrante di Ruffano et al.^27^ found that multispectral imaging achieved 92.9% sensitivity but only 43.6% specificity; in a hypothetical cohort of 1000 lesions at 20% melanoma prevalence, this would generate 451 false-positive results while missing 14 melanomas. Multiple reviews concluded that poor study quality and lack of real-world validation limit AI systems to a supportive role in specialist diagnostic decision-making.

Two reviews evaluated AI for histopathological melanoma diagnosis^36,37^. Clarke et al.^36^ reported pooled sensitivity of 90% and specificity of 92%, though study-level AUC ranged widely from 0.68 to 0.98 and studies using melanoma-only datasets reported higher accuracy than those including multiple diagnoses. Mosquera-Zamudio et al.^37^ reviewed 28 studies with similarly wide-ranging performance (accuracy 59–99.9%, sensitivity 48–97%, specificity 64–99%). Both reviews highlighted Al’s potential to reduce inter-observer variability but noted the absence of defined clinical workflows and prospective validation studies (Supplementary Figure S3).

Twenty-two reviews either spanned multiple screening stages^14,38–45^ or focused on algorithmic benchmarking^12,19,46–56^. Reported performance varied substantially (accuracy 40.0–100%, sensitivity 14.9–100%, specificity 12.0–100%, AUC 0.50–1.00) (Supplementary Figure S4) with substantial heterogeneity (I^2^ >75% where reported), limiting clinically actionable conclusions.

#### Human-AI collaboration (Q1.b)

Three reviews evaluated AI augmentation of clinician performance (Figure 3b)^14,40,43^. Krakowski et al.^40^, found AI assistance improved clinician sensitivity from 74.8% to 81.1% and specificity from 81.5% to 86.1%. Salinas et al.^14^ reported similar improvements (sensitivity 79.8% to 87.0%, specificity 73.6% to 77.1%), with Miller et al.^43^ finding AI assistance improved accuracy in melanoma detection using TBP by 15%. The benefit varied substantially by expertise: AI augmentation improved generalist sensitivity by 27.9% (64.6% to 92.5%) versus 2.1% for expert dermatologists (84.2% to 86.3%), though generalist specificity decreased by 6.3%, indicating increased false positives. By contrast, expert dermatologists maintained or improved specificity with AI assistance. All studies used curated images in experimental settings rather than consecutive clinical populations.

#### Performance by cancer type and imaging modality (Q1.c)

AI performance varied by cancer type (Figure 3c). Jones et al.^16^ found AI sensitivity for SCC was 60.3%, substantially lower than for melanoma (84.2%) or BCC (83.7%), indicating algorithms miss approximately 40% of SCCs. Melanoma, representing less than 2% of skin cancers^57^, was the exclusive focus of 15 of 37 reviews (40.5%) compared with only three (8.1%) addressing NMSC, which accounts for over 80% of skin cancer diagnoses^58^.

Imaging modality substantially influenced performance. Ferrante di Ruffano et al.^27^ found dermoscopy-based CAD achieved a sensitivity of 90.1% and specificity of 74.3%, while multispectral imaging attained 92.9% sensitivity but only 43.6% specificity. Patel et al.^31^ reported optical coherence tomography (OCT) achieved 95% accuracy for melanoma compared with 82.7% for RCM. However, no reviews reported performance stratified by both cancer type and imaging modality simultaneously, precluding the determination of whether differences reflect cancer characteristics, imaging technology, or their interaction. The strongest evidence supports melanoma detection using dermoscopy, while NMSC remains inadequately validated across all modalities.

### Population representativeness and equity (Q2)

#### Skin tone and ethnicity representation in reviews (Q2.a)

**Figure 4:**
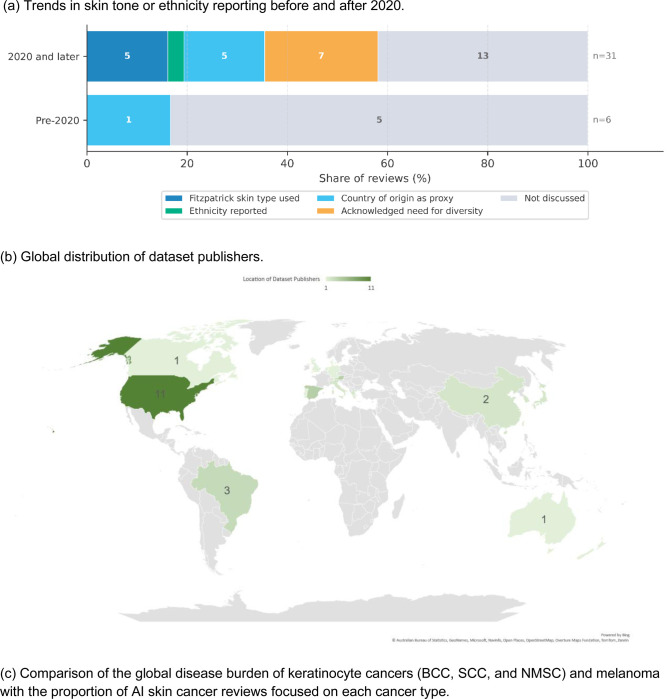

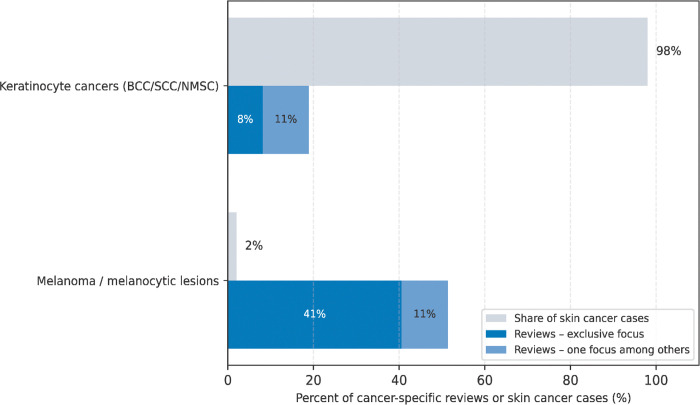
Population representativeness gaps in AI skin cancer research, showing (a) skin tone reporting over time, (b) dataset publisher geography, and (c) cancer type focus versus disease burden.

Among the 37 included reviews, 19 (51.4%) did not address ethnicity or skin tone in their analyses, while another seven (18.9%) discussed the need for diverse datasets without further analysis (Supplementary Figure S5). Only four reviews (10.8%) analyzed reporting of Fitzpatrick skin type, while six more (16.2%) reported country of origin as a proxy indicator. This gap has improved only moderately over time: among reviews published before 2020, five of six (83.3%) omitted demographic information; from 2020 onward, 21 of 31 reviews (67.7%) either discussed the need for diverse datasets without further analysis or omitted the discussion entirely (Figure 4a).

Reviews that addressed diversity revealed a measurement gap: studies with diverse populations rarely reported stratified performance. Liu et al.^41^ identified 22 studies with at least 10% skin-of-color representation in training datasets, yet none reported performance stratified by Fitzpatrick type. Steele et al.^19^ found only 1 of 114 studies (0.9%) reported performance across all skin phototypes. Jairath et al.^30^ similarly found that, among 232 studies, few provided stratified performance data despite identifying skin tone concerns.

#### Dataset characteristics and geographic distribution (Q2.b)

Analysis of reviews identified 35 publicly available datasets (Table 1; Supplementary Figure S6). Geographic concentration was pronounced (Figure 4b): >70% of dataset contributors originated from North America, Europe, or Oceania. The ISIC archive, cited in 21 reviews, was the only international collaborative dataset (56.8%), but lacks systematic skin phototype annotation. Dataset reuse was extensive, with ISIC-derived datasets appearing in over half of reviews, raising concerns that reported performance may reflect overlapping validation sets rather than independent populations. Only three datasets explicitly included skin tone annotations.

#### Evidence distribution and clinical disease burden alignment (Q2.c)

The distribution of evidence across cancer types was substantially misaligned with clinical disease prevalence (Figure 4c). Melanoma was the exclusive focus of 15 reviews (40.5%) and was evaluated alongside other cancers in four additional reviews (10.8%) (Supplementary Figure S7). By contrast, NMSC, which represents approximately 80% of skin cancer diagnoses^58^, was exclusively addressed in only four reviews (10.8%). Among identified datasets, diagnostic labels were heavily skewed toward melanoma and common benign lesions (nevi, seborrheic keratoses), while rare malignancies and inflammatory conditions remained substantially underrepresented, further compounding the evidence-practice gap (Supplementary Figure S8).

### Clinical implementation readiness and translational barriers (Q3)

**Figure 5:**
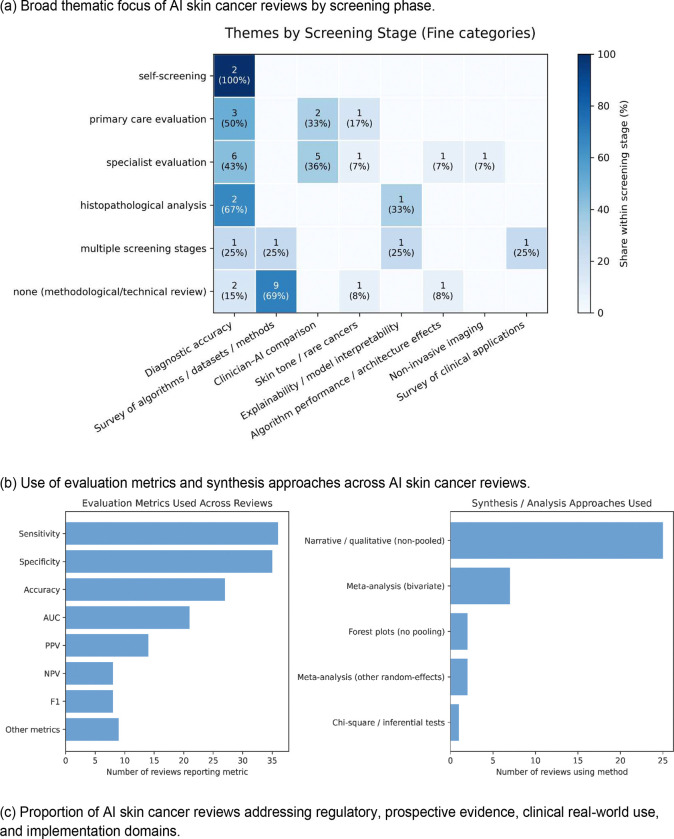

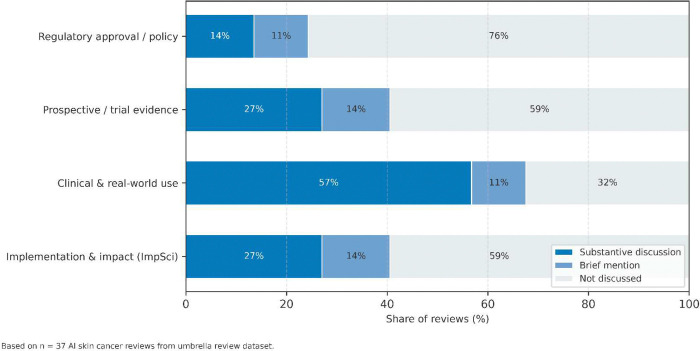
Implementation readiness and translational gaps, showing (a) research priorities by screening phase, (b) evaluation metrics and synthesis methods, and (c) review engagement with regulatory, validation, and implementation requirements.

Analysis of translational requirements revealed substantial gaps between technical performance reporting and implementation readiness (Figure 5).

#### Research priorities and evolution (Q3.a)

Among the 37 reviews, diagnostic accuracy was the predominant research focus (14 reviews, 37.8%), followed by algorithmic or dataset surveys (12 reviews, 32.4%) (Figure 5a; Supplementary Figure S9). Only one review examined AI model interpretability, and none systematically evaluated calibration techniques or uncertainty quantification. Research priorities remained heavily weighted toward technical performance assessment rather than clinical translation, with no meaningful shift over time.

#### Methodological characteristics (Q3.b)

Sensitivity and specificity were reported in 34 (91.9%) and 33 (89.2%) reviews, respectively (Figure 5b; Supplementary Figure S10), but PPV appeared in only 13 (35.1%) and NPV in 8 (21.6%), despite their importance for clinical decision-making in low-prevalence screening settings^59^. Advanced metrics were similarly scarce, with AUC appearing in 20 reviews (54.1%) and F1-score in 8 (21.6%). Methodological and technical reviews relied almost exclusively on qualitative synthesis (11 of 13, 84.6%), while clinical reviews more frequently attempted quantitative pooling (7 of 24, 29.2%).

#### Engagement with implementation requirements (Q3.c)

Only 5 of 37 reviews (13.5%) substantively discussed regulatory approval or device clearance pathways, while 28 (75.7%) made no mention of regulatory status (Figure 5c). No reviews systematically compared approval requirements across jurisdictions or assessed whether marketed systems met evidentiary standards.

Real-world deployment received extensive discussion (25 of 37 reviews, 67.6%), addressing workflow integration, performance concerns in routine clinical use, and safety risks. However, this discussion was largely theoretical: prospective validation in consecutive clinical populations remained rare, and no reviews identified studies assessing performance after clinical implementation. Reader studies represented the closest approximation to workflow testing but used curated datasets in controlled conditions.

Systematic engagement with implementation science frameworks was absent: no reviews employed RE-AIM, CFIR, or equivalent structured frameworks. Among 16 reviews (43.2%) that addressed implementation factors, discussion was exploratory rather than systematic. Five noted factors related to cost analysis, acknowledging that unnecessary biopsies impose economic costs, but none performed formal evidence synthesis.

Clinician acceptance and trust emerged as recurring themes^35,39,41^, with multiple specialist reviews discussing transparency in algorithmic decision-making as a tool to build clinician confidence. Patient perspectives, workforce training, organizational readiness, and technical infrastructure received minimal attention.

## Discussion

This umbrella review synthesized 37 systematic reviews to evaluate AI’s diagnostic performance, population representativeness, and implementation readiness across the skin cancer screening pathway. Three principal findings emerged: First, AI performance varies substantially across screening contexts: self-screening applications exhibit variability that poses safety risks, whereas specialist dermoscopy and histopathology achieve performance comparable to dermatologists. Second, equity gaps undermine generalizability, with engagement in skin tone reporting increasing after 2020 but remaining largely superficial, >70% of datasets originating from predominantly light-skinned populations, and evidence concentrated disproportionately on melanoma. Third, the evidence base prioritizes algorithmic benchmarking over clinical translation, with minimal engagement with regulatory pathways, clinical utility metrics, or implementation science frameworks.

Variations in AI performance across screening contexts have direct clinical implications. Self-screening applications, given their commercial availability and direct-to-consumer marketing, may provide false reassurance and delay evaluation of dangerous lesions. In specialist settings, AI approaches expert-level performance but has been validated predominantly on curated datasets, supporting its use only as a decision support tool. AI augmentation benefits generalists most substantially. This suggests the greatest potential benefit in primary care settings where expertise is limited, though the increased false-positive rates among generalists require appropriate referral thresholds. Finally, the performance gap for SCC detection (60.3% vs 84.2% for melanoma) warrants particular vigilance, as systems are optimized for the rarest, deadliest skin cancers while underperforming on those clinicians encounter most frequently.

The equity gaps identified raise questions about which populations current AI tools can reliably serve. While post-2020 reviews show increased engagement with skin tone and ethnicity, this progress is largely rhetorical; the growth is concentrated in acknowledgements of diversity needs rather than systematic characterization of training data or stratified reporting of outcomes. More concerning than dataset composition is the persistent failure to report stratified performance; even among studies intentionally including diverse populations, none reported results by Fitzpatrick type^41^, meaning the magnitude of potential algorithmic inequity remains unknown. Until the field prioritizes both diverse validation datasets and mandatory stratified reporting, AI tools validated primarily on light-skinned populations should not be assumed to perform equivalently across all patient groups.

The translational barriers identified reflect structural features in how the field generates evidence. A minority of reviews addressed regulatory approval, despite some CE-certified smartphone applications demonstrating poor melanoma detection performance. While almost all reviews discussed deployment considerations, these discussions were largely theoretical, drawing implications from retrospective validation inadequate for predicting real-world performance. Addressing this gap will require prospective, workflow-embedded evaluations that assess AI performance across consecutive clinical cases, measure downstream outcomes such as biopsy rates and diagnostic delays, and capture implementation factors, including clinician trust and workflow integration. The absence of implementation science frameworks suggests the field remains oriented toward algorithmic benchmarking rather than clinical translation.

Our findings complement the recent umbrella review by Karimzadhagh et al.^23^, which synthesized 11 meta-analyses and shares 8 sources with our review^14,16,26,27,32,35,40,52^. Their conclusions regarding diagnostic accuracy are broadly consistent with ours: melanoma models typically achieve >85% sensitivity, SCC detection lags substantially, and AI assistance benefits generalists more than expert dermatologists. Their analysis of algorithm architecture and validation methodology provides technical depth that complements our focus. Whereas Karimzadhagh et al. addressed whether AI works, our pathway-based synthesis of 37 reviews asks for whom, under what conditions, and why deployment has not occurred, revealing setting-specific safety concerns, equity gaps, and translational barriers that have not been previously addressed in umbrella reviews.

This review has several limitations. We did not exclude reviews based on quality, meaning high-risk-of-bias reviews contribute to our synthesis alongside methodologically stronger ones, though our ROBIS assessment transparently reports this variation. The ROBIS tool, while validated for systematic reviews broadly, is not specific to AI diagnostic accuracy studies and may not capture domain-specific concerns such as data leakage or inappropriate performance benchmarks. Because umbrella reviews synthesize already aggregated evidence, we could not assess overlap at the primary study level. Given the dominance of ISIC-derived datasets across reviews, substantial data overlap is likely, potentially inflating the apparent consistency of findings. We were unable to conduct a meta-analysis, as pooling estimates derived from reviews that may themselves have pooled overlapping primary studies would compound aggregation bias and yield misleadingly precise estimates. Finally, the rapid pace of AI development means recent algorithmic advances may not yet be captured in published systematic reviews.

Several strengths balance these limitations. We synthesized evidence spanning the entire screening pathway using dual screening, ROBIS assessment, and PRISMA adherence. Organizing by screening phase revealed context-specific safety concerns not apparent in aggregate analyses. Our integration of equity and implementation domains provides the most comprehensive assessment to date of where AI dermatology tools are advancing and where fundamental evidence gaps remain.

These findings have implications for multiple stakeholders. Clinicians should counsel patients against relying on self-screening applications and consider AI as decision support only, with particular vigilance for SCC. Developers must report performance stratified by Fitzpatrick skin type, prioritize NMSC validation, and report PPV and NPV alongside sensitivity and specificity. Regulators should establish stage-specific validation standards with stringent requirements for patient-facing tools. Researchers must prioritize prospective validation in consecutive clinical populations and evidence on BCC and SCC commensurate with their disease burden. Without coordinated efforts across these domains, AI dermatology risks perpetuating existing healthcare inequities while providing suboptimal benefit to the populations most in need of accessible diagnostic support.

## Methods

We conducted this umbrella review following PRISMA guidelines^60^. The protocol was prospectively registered in PROSPERO (CRD42024605934). Data extraction templates and results can be requested from the corresponding author. Generative AI (Claude, Anthropic, 2024–2025) was used to assist with manuscript editing, including grammar, language refinement, and formatting compliance. All AI-assisted outputs were reviewed, verified, and revised by the authors, who take full responsibility for the accuracy and integrity of the work.

### Search strategy and selection criteria

We searched PubMed, Web of Science, and CINAHL databases on November 6th, 2024, with no date or language restrictions. We included systematic reviews and meta-analyses evaluating AI for skin cancer detection across any care setting, as well as methodological reviews focused on algorithm development and datasets. Studies were required to report at least one diagnostic accuracy metric. We excluded individual primary studies, scoping reviews, narrative reviews, and reviews not focused on diagnostic accuracy. Full search strategies and inclusion criteria are in Supplementary Table S2.

Two investigators (L.S., A.B.) independently screened titles, abstracts, and the full texts, with disagreements resolved through discussion. Studies excluded at full-text review with reasons are listed in Supplementary Table S3.

### Data collection and quality assessment

One investigator (L.S.) extracted data with verification by a second (A.B.). We extracted screening pathway phase, cancer types, imaging modalities, diagnostic performance, skin tone representation, and implementation science considerations (Table 2); additional methodological characteristics, including AI architectures, protocol registration, and authors’ countries of origin, were extracted for quality assessment and are reported in Supplementary Table S1. Both investigators independently assessed risk of bias using Risk of Bias in Systematic Reviews (ROBIS)^61^ across four domains: study eligibility criteria, identification and selection, data collection and appraisal, and synthesis and findings (Supplementary Table S4). Missing data are annotated accordingly in the extraction chart; study investigators were not contacted. Microsoft Excel was used to facilitate data extraction and management.

### Data synthesis

Given substantial heterogeneity across AI tasks, datasets, and validation methods, we synthesized findings narratively, following SWiM guidelines^62^, rather than conducting a meta-analysis. We grouped reviews by screening pathway phase and reported performance metrics as ranges across reviews.

We classified dataset source countries as predominantly light-skinned (Europe, North America, Oceania) or skin-of-color (South/East Asia, Middle East, North Africa, Latin America) populations based on typical regional phototype distributions^63^; acknowledging this classification represents a simplification of within-region heterogeneity.

### Changes from protocol

This review followed the registered protocol (PROSPERO CRD42024605934). To enhance clinical relevance, we organized our synthesis around three domains, addressing questions prioritized by clinicians and policymakers: diagnostic performance across care settings, population representativeness and equity, and implementation readiness. This framework addresses the same data elements specified in the protocol while emphasizing actionable insights.

## Supplementary Material

This is a list of supplementary files associated with this preprint. Click to download.

- UmbrellaReviewSupplementaryMaterial3826.docx

## Figures and Tables

**Table 1:** Publicly available and named or available-upon-request dermatology datasets identified in the included reviews.

Title	ISIC	Number of Reviews	Image Type	Number of Samples	Class Labels	Public Accessibility	Year Published	Location of Dataset Publishers	Countries of Origin of Skin Samples	Skin Tone Annotations
Asan & Hallym	No	5	macroscopic	17125	NV - 2706, SK - 1423, DF - 1247, SCC - 1231, SL - 1193, BCC - 1082, IC - 918, AKIEC - 651, MM - 599, PG - 375, wart - 2985, HAE - 2715	on request	2017	South Korea	South Korea	No
Atlas Dermatologico	No	4	macroscopic	12644	588 skin conditions	yes	1999	Brazil	Brazil	No
BCN20000	Yes	6	dermoscopic	18946	NV, MM, BCC, SK, AK, SCC, DF, VL, Other	yes	2019	Spain	Spain	No
CSID	No	2	dermoscopic & macroscopic	not publicly release d	various skin conditions	no	2017	China	China	No
DanDerm	No	4	macroscopic	3000	various skin conditions	yes	2003	Denmark	Denmark	No
Derm7pt	Yes	5	dermoscopic	2000	MM, BCC, SK, DF, SL, VL	yes	2018	Canada, Italy	not stated - likely Canada	No
DermAtlas	No	2	macroscopic	1000	various skin conditions	yes	2003	USA	USA	No
Dermatoweb	No	1	macroscopic	7300	various skin conditions	yes	2009	Spain	Spain	No
DermlS	No	8	macroscopic	6588	various skin conditions	yes	2000	Germany	Germany	No
Dermnet	No	7	dermoscopic & macroscopic	23000	various skin conditions	yes	1998	USA	not stated - likely USA	No
Dermnet NZ	No	2	macroscopic	20000	more than 600 skin conditions (unknown proportion of skin cancer)	partially	1996	New Zealand	>10 countries, NZ-led	Yes
Dermquest	No	6	dermoscopic	22082	various skin conditions	no longer available	1999	USA	not stated	No
Edinburgh Dermofit Library	No	5	macroscopic	1300	AK - 45, BCC - 239, NV - 331, SK - 257, SCC - 88, AKIEC - 78, PG - 24, DF - 65, HMG - 97, MM - 76	for purchase	2013	UK	UK	No
EDRA-CDROM	No	5	dermoscopic	1024	AK - 5, BCC - 42, BK - 70, DF - 20, NV - 275, MM -, 582, VL - 30	for purchase	2000	Italy	Italy & other European countries	No
Hellenic Derm Atlas	No	4	macroscopic	2660	various skin conditions	yes	2010	Greece	Greece	No
HAM10000	Yes	18	macroscopic	11720	AK - 327, BCC - 514, SK - 1099, DF - 115, MM - 1113, NV - 6705, VL - 142	yes	2018	Austria	Australia, Austria	No
HIAE	No	1	macroscopic	5267	PC (pigmentati on changes), ID (inflammatory diseases), SL (suspicious lesions), BT (benign tumors)	on request	2021	Brazil	Brazil	No
ISIC archive/unspecified ISIC dataset	Yes	21	macroscopic & macroscopic	503955	MM, NV, BCC, AK, BK, DF, VL, SK, Lentigo, Other Benign Lesions, SCC, AKIEC, PG, HMG, Cutaneous Metastases	yes	2016	International	multicountry	Partial
ISIC 2016	Yes	11	macroscopic	1279	MM, NV	yes	2016	USA	USA, Europe	No
ISIC 2017	Yes	12	macroscopic	2750	MM, NV, SK	yes	2017	USA, Austria	USA, Austria	No
ISIC 2018	Yes	11	macroscopic	15414	MM, NV, BCC, AKIEC, BK, DF, VL, Benign vs Malignant	yes	2018	USA, Austria	USA, Austria, Australia	No
ISIC 2019	Yes	8	macroscopic	36313	M, BCC, AK, SCC, SK, DF, NV, VL	yes	2019	USA, Austria, Spain	USA, Austria, Spain	No
ISIC 2020	Yes	5	macroscopic	44108	NV, MM, SK	yes	2020	USA, Australia, Greece, Spain	USA, Australia, Greece, Spain	No
KCGMH	No	2	macroscopic	1287	BCC, SCC, MM, BK, NV	on request	Unknown	Taiwan	Taiwan	No
Meddean	Yes	1	macroscopic & histological	300	232 skin conditions	yes	1995	USA	USA	No
MED-NODE	Yes	11	macroscopic & macroscopic	170	MM - 70, SK - 100	yes	2015	Netherland s	Netherlands	No
MSK	Yes	2	macroscopic	10982	MM, NV, BCC, AK, BK, DF, VL, SCC	yes	2018	USA	USA	Yes
NSDD	No	1	macroscopic	not publicly released	not publicly released	no	Unknown	Japan	Japan	No
PAD-UFES-20	Yes	1	macroscopic	2298	NV - 244, MM- 52, BCC - 845, BKL, AKIEC - 730, SCC - 192, SK - 235	yes	2020	Brazil	Brazil	Yes
PH2	No	18	macroscopic	200	NV - 80, Atypical NV - 80, MM - 40	yes	2013	Portugal	Portugal	No
Shinshu	Partial	4	0	not publicly release d	MM, SK, NV	no	Unknown	Japan	Japan	No
SD-198	No	1	macroscopic	6584	various skin conditions	yes	2016	USA	not stated	No
SNU	No	4	0	2201	134 skin conditions	on request	2024	South Korea	South Korea	No
XiangyaDerm	No	1	macroscopic	150000	571 skin conditions	no	2021	China	China	No

**Table 2:** Characteristics of included systematic reviews.

Reference	Cancer Type Targeted	Imaging Modality	Population / Skin Tone Discussion	Diagnostic Performance	Implementation Considerations
**Self Screening**					
Chuchu, 2018^24^	Melanoma	Commercial smartphone/camera	not discussed	Sensitivity range: 7–73% Specificity range: 37–94%	Regulatory approval: N Prospective studies: S Real-world deployment: S Implementation science: N
Freeman, 2020^25^	Any	Commercial smartphone/camera	not discussed	Sensitivity range: 0–97% Specificity range: 19–100%	Regulatory approval: S Prospective studies: S Real-world deployment: S Implementation science: S
**Primary Care**					
Abdalla, 2024^26^	Suspicious Pigmented Lesions (SPLs)	clinical macroscopic	not discussed	Pooled sensitivity: 90% Pooled specificity: 85% Pooled AUC: 0.95	Regulatory approval: N Prospective studies: S Real-world deployment: S Implementation science: S
Jones, 2022^16^	Melanoma, BCC, SCC	clinical macroscopic	Fitzpatrick skin type	Pooled accuracy: - MM: 89.5% - SCC: 85.3% - BCC: 87.6% - NV vs MM: 88.8% Pooled sensitivity: - MM: 84.2% - SCC: 60.3% - BCC: 83.7% - NV vs MM: 87.0% Pooled specificity: - MM: 89.1% - SCC: 93.3% - BCC: 88.7% - NV vs MM: 86.4% Pooled AUC: - MM: 0.898 - SCC: 0.875 - BCC: 0.923 - NV vs MM: 0.883	Regulatory approval: S Prospective studies: S Real-world deployment: S Implementation science: S
**Specialist Evaluation**					
Ferrante di Ruffano, 2018^27^	Melanoma, BCC, SCC	dermoscopic, multispectral imaging, spectroscopy	not discussed	Pooled sensitivity: - Derm-CAD: 90.1% - MSI-CAD: 92.9% Pooled specificity: - Derm-CAD: 74.3% - MSI-CAD: 43.6%	Regulatory approval: N Prospective studies: S Real-world deployment: S Implementation science: N
Foltz, 2024^28^	NMSC	dermoscopic, RCM, HEI	discussed need for diverse datasets	Pooled accuracy: 86.8% Sensitivity range: 70.6–95.6% Specificity range: 41.8–100% AUC range: 0.80–0.99	Regulatory approval: N Prospective studies: N Real-world deployment: S Implementation science: S
Höhn, 2021^29^	Any	dermoscopic	not discussed	Accuracy range: 61.0–92.0% Sensitivity range: 52.7–87.6% Specificity range: 90.2–91.0%	Regulatory approval: N Prospective studies: N Real-world deployment: N Implementation science: N
Jairath, 2024^30^	Melanoma, Keratinocyte Carcinoma	dermoscopic, clinical macroscopic	Fitzpatrick skin type	Pooled accuracy: 90.0% Pooled sensitivity: 87.0% Pooled specificity: 91.0%	Regulatory approval: S Prospective studies: S Real-world deployment: S Implementation science: S
Patel, 2023^31^	Melanoma	dermoscopic, OCT, RCM	discussed need for diverse datasets	Pooled accuracy: OCT: 95%, RCM: 82.72% Pooled sensitivity: 83.01% Pooled specificity: 85.58%	Regulatory approval: N Prospective studies: N Real-world deployment: N Implementation science: S
Rajpara, 2009^32^	Melanoma	dermoscopic	not discussed	Pooled sensitivity: 91.00% Pooled specificity: 79.00%	Regulatory approval: N Prospective studies: B Real-world deployment: S Implementation science: B
Vestergaard, 2008^33^	Melanoma	electrical impedance spectroscopy (EIS), dermoscopic, telespectrometry (TS)	not discussed	Pooled sensitivity: - clinician: 62–91% - instrument: 80–100% Pooled sensitivity: - clinician: 36–99% - instrument: 49–98%	Regulatory approval: S Prospective studies: B Real-world deployment: S Implementation science: N
Widaatalla, 2023^34^	BCC	dermoscopic, OCT, RCM	not discussed	Accuracy range: 81.0–99.0% Sensitivity range: 68.0–100.0% Specificity range: 85.0–100.0% AUC range: 0.86–0.99	Regulatory approval: N Prospective studies: N Real-world deployment: S Implementation science: N
Ye, 2024^35^	Melanoma	dermoscopic	discussed need for diverse datasets	Pooled sensitivity: 82.0% Pooled specificity: 87.0% Pooled AUC: 0.92	Regulatory approval: N Prospective studies: S Real-world deployment: B Implementation science: N
**Histopathological Analysis**					
Clarke, 2023^36^	Melanoma	histopathological (WSI)	not discussed	Accuracy range: 79.7–97.8% Pooled sensitivity: 90% Pooled specificity: 92% AUC range: 0.68–0.98	Regulatory approval: N Prospective studies: S Real-world deployment: S Implementation science: N
Mosquera-Zamudio, 2022^37^	Melanoma, melanocytic tumors	histopathological (WSI)	not discussed	No accuracy, sensitivity, specificity, or AUC information provided	Regulatory approval: N Prospective studies: N Real-world deployment: S Implementation science: S
**Multiple Screening Stages**					
Haggenmüller, 2021^38^	Melanoma	dermoscopic, clinical macroscopic, histopathological (WSI)	ethnicity, country of origin	Sensitivity range: - CNN: 49.5–80.5% - human clinicians/raters: 65.8–77.6% Specificity range: - CNN: 51.3% - 90.0% - human clinicians/raters: 85.7% - 95.6%	Regulatory approval: N Prospective studies: B Real-world deployment: S Implementation science: N
Hauser, 2022^39^	Any	any	not discussed	No accuracy, sensitivity, specificity, or AUC information provided	Regulatory approval: N Prospective studies: N Real-world deployment: S Implementation science: N
Krakowski, 2024^40^	Any	dermoscopic, clinical macroscopic	country of origin	Pooled sensitivity: - Clinicians + AI: 81.1% - Clinicians: 74.8% Pooled specificity: - Clinicians + AI: 86.1% - Clinicians: 81.5%	Regulatory approval: S Prospective studies: S Real-world deployment: S Implementation science: N
Liu, 2023^41^	Suspicious Pigmented Lesions (SPLs)	any	Fitzpatrick skin type, country of origin	Accuracy range: - Binary: 70.0–99.7% - Multiclass: 43.0–93.0% - Risk-category: 83.0% - Reader studies: (AI) 45.0–92.0%, (Expert) 60.0–90.0%, (Non-expert) 40.0–94.0% Sensitivity range: - Binary: 63.0–100.0% - Multiclass: 58.0–88.0% - Risk-category: 85.0–93.0% - Reader studies: (AI) 67.0–93.0%, (Expert) 61.0–97.0%, (Non-expert) 45.0–85.0% Specificity range: - Binary: 72.0–99.0% - Multiclass: 81.0–99.0% Risk-category: 85.0–91.0% - Reader studies: (AI) 72.0–95.0%, (Expert) 58.0–99.0%, (Non-expert) 46.0–94.0% AUC range: - Binary: 0.77–0.95 - Multiclass: Not consistently reported - Risk-category: 0.92–0.96 - Reader studies: (AI) 0.77–0.94, (Expert) 0.80–0.91, (Non-expert) 0.110.88	Regulatory approval: N Prospective studies: N Real-world deployment: B Implementation science: N
Marka, 2019^42^	NMSC	dermoscopic, clinical macroscopic	not discussed	Accuracy range: 72–100% Pooled sensitivity: 38.0–100% Pooled specificity: 12.0–100% AUC range: 0.83–1.00	Regulatory approval: N Prospective studies: B Real-world deployment: S Implementation science: B
Miller, 2024^43^	Melanoma	commercial smartphone/camera, 2D TBP, 3D TBP	country of origin	Accuracy range: - Mobile Applications: 62.3–92.0% - 3D TBP: 65.6–68.0% - 2D TBP: 44.0–44.3% - Clinicians + AI: 86.4–86.9% - CNN: 54.2–87.7% - Clinician, No AI: 52.0–92.0% Pooled sensitivity: - Mobile Applications: 80.0–92.8% - 3D TBP: 83.3–90.0% - 2D TBP: 70.0–83.3% - Clinicians + AI: 83.3–100.0% - CNN: 16.4–100.0% - Clinician, No AI: 41.8–96.6% Specificity range: - Mobile Applications: 60.0–95.0% - 3D TBP: 63.6–64.6% - 2D TBP: 40.0–40.0% - Clinicians + AI: 83.7–87.3% - CNN: 54.4–98.3% - Clinician, No AI: 32.2–92.7% AUC range: - Mobile Applications: 0.717 - 3D TBP: 0.92–0.94 - 2D TBP: 0.68 - Clinicians + AI: 0.88–0.968 CNN: 0.540–0.969 - Clinician, No AI: 0.778–0.91	Regulatory approval: N Prospective studies: S Real-world deployment: S Implementation science: S
Salinas, 2024^14^	Any	dermoscopic, clinical macroscopic	discussed need for diverse datasets	Pooled sensitivity: - AI (overall): 87.0% - Clinicians: 79.78% - AI vs Generalists: 92.5% - Generalists: 64.6% - AI vs Experts: 86.3% - Experts: 84.2% Pooled specificity: - AI (overall): 77.1% - Clinicians: 73.6% - AI vs Generalists: 66.5% - Generalists: 72.8% - AI vs Experts: 78.4% - Experts: 74.4%	Regulatory approval: B Prospective studies: S Real-world deployment: S Implementation science: S
Wu, 2022^45^	Any	any	discussed need for diverse datasets	Accuracy range: 62.2–99.1% Sensitivity range: 78.7–97.2% Specificity range: 81.2–98.1% AUC range: 0.67–0.98	Regulatory approval: N Prospective studies: N Real-world deployment: N Implementation science: N
Zhang, 2022^44^	Melanoma	any	not discussed	Accuracy range: 81.0–95.0% Sensitivity range: 69.3–100.0% Specificity range: 41.8–99.0% AUC range: 0.81–0.95	Regulatory approval: N Prospective studies: N Real-world deployment: N Implementation science: N
**Methodological/Technical Reviews**					
Brinker, 2018^46^	Any	any	country of origin, need for diverse datasets	Accuracy range: 50.3%−93.6% Sensitivity range: 58.0–93.1% Specificity range: 80.0–95.2% AUC range: 0.86–0.96	Regulatory approval: N Prospective studies: N Real-world deployment: B Implementation science: B
Dildar, 2021^47^	Any	any	country of origin	Accuracy range: 70.1–98.9% Sensitivity range: 14.9–95.2% Specificity range: 80–96.5% AUC range: 0.85–0.96	Regulatory approval: N Prospective studies: N Real-world deployment: N Implementation science: B
Furriel, 2024^48^	Any	dermoscopic, clinical macroscopic	country of origin, Fitzpatrick skin type	Accuracy range: 76.5–97.5% Sensitivity range: 60.7–100% Specificity range: 45.4–100% AUC range: 0.89–0.95	Regulatory approval: B Prospective studies: B Real-world deployment: S Implementation science: S
Grignaffini, 2022^49^	Any	dermoscopic	not discussed	Accuracy range: 86.0–100% Sensitivity range: 41.0%−100.0% Specificity range: 80.0%−100.0%	Regulatory approval: N Prospective studies: N Real-world deployment: N Implementation science: N
Hameed, 2024^50^	Any	dermoscopic	not discussed	Accuracy range: 76.1–99.4%	Regulatory approval: N Prospective studies: N Real-world deployment: N Implementation science: N
Hermosilla, 2024^51^	Any	any	country of origin	Pooled accuracy: 71.0% Pooled specificity: 53.0%	Regulatory approval: N Prospective studies: N Real-world deployment: B Implementation science: N
Li, 2022^52^	Melanoma	any	not discussed	Pooled sensitivity: 85.0% Pooled specificity: 86.0% Pooled AUC: 0.87	Regulatory approval: N Prospective studies: N Real-world deployment: N Implementation science: B
Lyakhova, 2024^12^	Any	any	not discussed	Accuracy range: 56.52–98.42%	Regulatory approval: N Prospective studies: N Real-world deployment: S Implementation science: N
Mazhar, 2023^53^	Any	clinical macroscopic, commercial smartphone/camera, dermoscopic	not discussed	Accuracy range: 80.5% - 99.0% Sensitivity range: 52.5–100.0% Specificity range: 80.5–99.1%	Regulatory approval: N Prospective studies: N Real-world deployment: N Implementation science: N
Naeem, 2020^54^	Melanoma	dermoscopic	discussed need for diverse datasets	Accuracy range: 80.5–99.5% Sensitivity range: 49.0–100.0% Accuracy range: 84.0–99.2% AUC range: 0.50–0.99	Regulatory approval: N Prospective studies: N Real-world deployment: N Implementation science: N
Popescu, 2022^55^	Melanoma	any	not discussed	Accuracy range: 72.3–97.5%	Regulatory approval: N Prospective studies: N Real-world deployment: N Implementation science: N
Riaz, 2023^56^	Melanoma, NMSC	any	discussed need for diverse datasets	Accuracy range: 87.00–99.92% AUC range: 0.80–0.98	Regulatory approval: N Prospective studies: S Real-world deployment: N Implementation science: N
Steele, 2023^19^	Rare skin cancers	dermoscopic, clinical macroscopic	country of origin	Accuracy range: 50.8–95.4% Sensitivity range: 52.3–72.6%	Regulatory approval: N Prospective studies: N Real-world deployment: S Implementation science: S

## Data Availability

The systematic review protocol is registered with PROSPERO (CRD42024605934). Data extraction tables, risk of bias assessments, and search strategies are available in the supplementary appendix. Study-level data extracted from included systematic reviews are available from the corresponding author upon reasonable request. The dataset does not contain individual patient data.

## Code Availability

No custom analytical code was developed for this study. Figures were generated using standard Python libraries (matplotlib, pandas, seaborn). Figure-generation scripts are available from the corresponding author upon reasonable request.
